# Effects of statins on sarcopenia with focus on mechanistic insights and future perspectives

**DOI:** 10.3389/fphar.2025.1669591

**Published:** 2025-09-30

**Authors:** Xianxu Zhang, Xianghong Wang, Lei Huang, Changlin Zhou, Bin Qian, Zhiqiang Luo, Yanqiang Chen

**Affiliations:** ^1^ Department of Orthopaedics, Lanzhou University Second Hospital, Lanzhou, Gansu, China; ^2^ Orthopaedics Key Laboratory of Gansu Province, Lanzhou University Second Hospital, Lanzhou, Gansu, China; ^3^ Gansu Jiuquan Hospital of Traditional Chinese Medicine, Jiuquan, Gansu, China

**Keywords:** sarcopenia, statins, inflammation, oxidative stress, mitochondrial function, drug pleiotropy

## Abstract

Sarcopenia is an age-related geriatric syndrome characterized by the progressive decline in skeletal muscle mass and function. Its pathogenesis is multifactorial, involving complex interactions between inflammatory responses, oxidative stress, and mitochondrial dysfunction. In recent years, statins, widely used as lipid-lowering agents, have garnered attention for their pleiotropic biological effects, particularly in their potential therapeutic value for anti-inflammatory, antioxidant, microcirculation improvement, and regulation of skeletal muscle metabolism. Statins have demonstrated promising potential in the prevention and treatment of sarcopenia. This review systematically examines the mechanisms through which statins act in sarcopenia, highlighting their pharmacological properties, biological effects, and relevant clinical and preclinical research advancements. Existing studies suggest that moderate statin use may improve the skeletal muscle microenvironment and maintain mitochondrial homeostasis by inhibiting the NF-κB signaling pathway, activating the Nrf2/ARE pathway, and modulating the AMPK/SIRT1/PGC-1α pathways. However, evidence also indicates that high doses or statin use in genetically predisposed individuals may result in mitochondrial dysfunction and muscle toxicity. Based on these findings, this paper proposes a “dose-mechanism-individual background” three-dimensional interaction model and further explores its potential synergistic or antagonistic effects when combined with exercise and nutritional interventions. Future research should prioritize conducting prospective clinical trials with stratified designs to develop individualized, precise intervention strategies, thus advancing the translational application of statins in managing muscle health in aging populations.

## 1 Introduction

Sarcopenia is a syndrome closely associated with aging, characterized by the progressive decline in skeletal muscle mass, strength, and function. As the global population continues to age, sarcopenia has emerged as one of the most pressing public health concerns, posing significant risks to the health and well-being of the elderly population (Wang et al., 2025). Epidemiological studies suggest that the global prevalence of sarcopenia varies between approximately 10% and 23%, with the prevalence in individuals aged 60 and above estimated at around 10%. Notably, more than half of individuals aged 75 and older are affected by varying degrees of sarcopenia (Petermann-Rocha et al., 2022; Shafiee et al., 2017; Köller, 2023). Additionally, growing evidence has shown that sarcopenia significantly increases the likelihood of adverse outcomes, including falls, fractures, hospitalization, and all-cause mortality (Janssen et al., 2002; Bosaeus and Rothenberg, 2016). Despite its widespread impact, effective treatment options for sarcopenia remain limited, and no pharmacological interventions have been specifically approved by regulatory authorities. Current treatment strategies primarily focus on exercise regimens and nutritional supplementation, such as resistance training, protein intake, and vitamin D supplementation. However, for elderly patients with multiple comorbidities and restricted mobility, the efficacy of these interventions is often suboptimal. Consequently, there is an urgent need for the development of novel pharmacological interventions to improve treatment outcomes for this vulnerable population (Calvani et al., 2023; Ganapathy and Nieves, 2020; Li et al., 2021).

Recent years have witnessed growing attention towards statins, a widely prescribed class of lipid-lowering agents, owing to their pleiotropic effects. Beyond their primary function of regulating cholesterol metabolism, statins demonstrate a diverse array of biological effects, including anti-inflammatory, antioxidant, immune-modulatory, endothelial function enhancement, promotion of bone formation, and inhibition of bone resorption (Shishehbor et al., 2003; Wang et al., 2008; Granat et al., 2024). Accumulating evidence suggests that statins may hold promise for the prevention and treatment of sarcopenia through mechanisms such as the improvement of skeletal muscle metabolism, preservation of mitochondrial function, and modulation of the local muscle microenvironment (Zheng et al., 2023; Liu et al., 2019). However, some studies have reported that, particularly in certain populations—especially those using high doses or specific statin formulations—these medications may exacerbate muscle toxicity and even provoke adverse muscle events (Muntean et al., 2017; Harper and Jacobson, 2007). Such discrepancies are likely influenced by variables such as drug type, dosage, individual susceptibility, and study design.

Given the substantial heterogeneity in these findings, it is imperative to systematically examine the potential biological mechanisms and clinical applications of statins in the context of sarcopenia. This review aims to provide an overview of the current state of research, focusing on the mechanisms by which statins exert effects on inflammation, oxidative stress, mitochondrial function, and bone metabolism regulation. Furthermore, it will evaluate the differential impacts of various statin types and dosages on sarcopenia and explore their therapeutic potential and safety in specific patient populations, considering individual metabolic profiles. The article will sequentially present a general overview of sarcopenia, the pharmacological properties of statins and their pleiotropic mechanisms, recent advances in related therapeutic interventions, and will discuss the limitations of current research and propose directions for future studies.

## 2 Sarcopenia

### 2.1 Prevalence

Sarcopenia is an age-related syndrome characterized by the progressive decline in skeletal muscle mass and function. In recent years, it has attracted significant attention in the context of global population aging. Several prominent international academic organizations, including ESPEN, EWGSOP, IWGS, SCWD, AWGS, FNIH, and SDOC, have proposed diagnostic criteria aimed at standardizing the identification and management of this condition, thereby emphasizing its growing importance in the field of geriatric medicine. Due to variations in assessment parameters and diagnostic thresholds, the reported prevalence of sarcopenia in different studies exhibits substantial discrepancies. According to the EWGSOP criteria, the prevalence of sarcopenia in the Asian population is approximately 21%, while in the European population, it is 22%. Under the AWGS criteria, the prevalence in Asia is 15%, whereas in Europe, it can rise to as high as 33% (Cruz-Jentoft et al., 2019; Chiu et al., 2025; Chen et al., 2020). Longitudinal studies have demonstrated a consistent decline in muscle mass among individuals under the age of 75, with the annual muscle loss rate ranging from 0.8% to 0.98% in males and from 0.64% to 0.70% in females. In contrast, the rate of muscle strength decline is even more pronounced: at the age of 75, males lose an average of 3%–4% of muscle strength per year, and females lose 2.5%–3%, significantly increasing the risk of falls and functional impairments (Mitchell et al., 2012; Wilkinson et al., 2018). Prevalence rates for sarcopenia differ markedly across various populations: in elderly individuals living in the community, the prevalence is 11% in males and 9% in females; in hospitalized patients, it increases to 23% in males and 24% in females; and in long-term care institutions, it reaches 51% in males and 31% in females (Papadopoulou et al., 2020). In multiple studies conducted in China, the prevalence in community populations ranges from 11% to 19%, while in Thailand, the prevalence is reported to be 18.1% (Liu et al., 2020; Hai et al., 2017; Yang et al., 2018; Asavamongkolkul et al., 2024). Furthermore, individuals with chronic conditions, such as diabetes, face a significantly elevated risk of developing sarcopenia (Yuan and Larsson, 2023). In conclusion, sarcopenia has emerged as one of the major geriatric syndromes that urgently require global attention, presenting a critical public health challenge.

### 2.2 Pathogenesis

**FIGURE 1:**
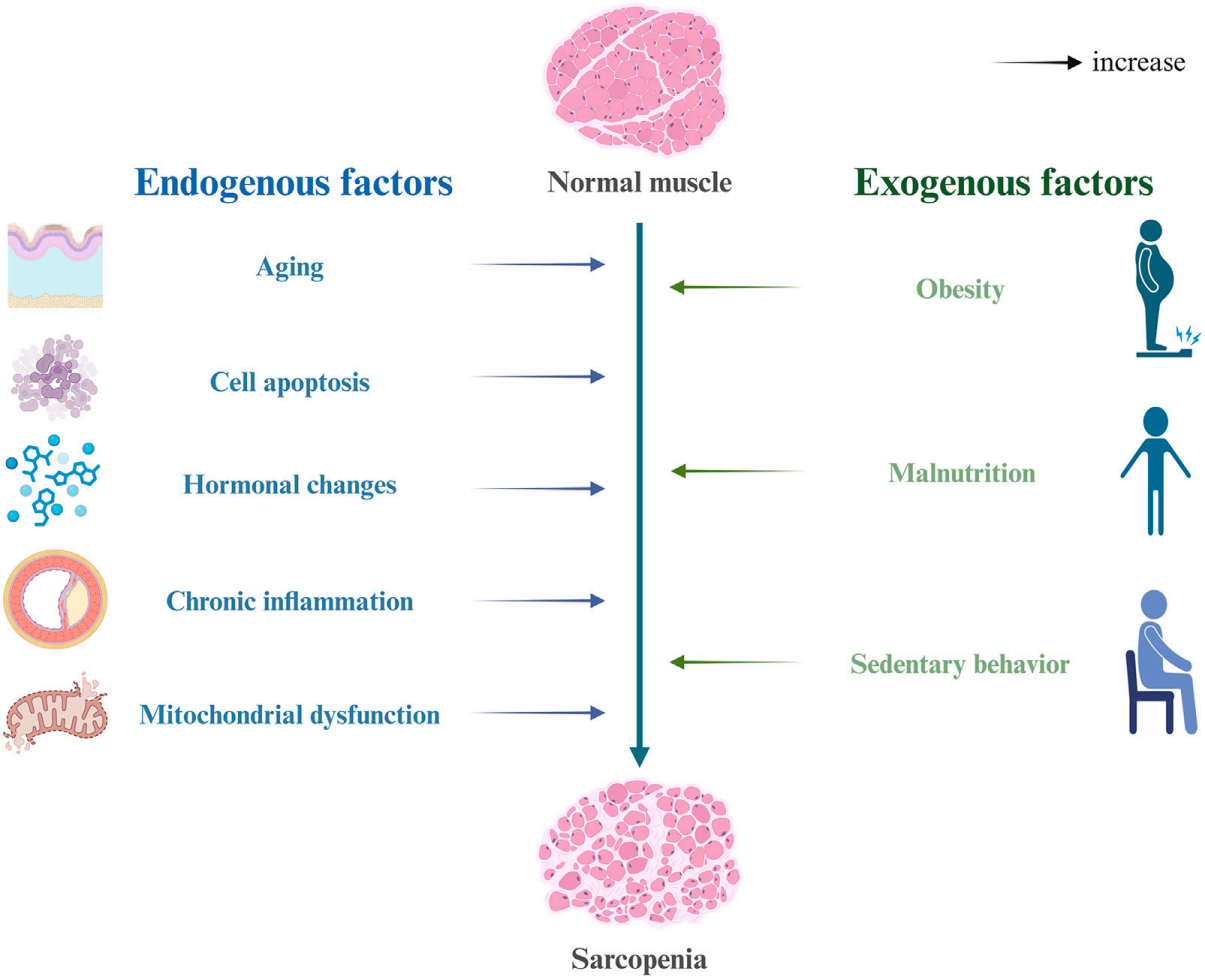
Risk factors and pathogenesis of sarcopenia.

Sarcopenia is a degenerative condition driven by the interaction of multiple factors. Although its pathogenesis remains incompletely understood, it is widely acknowledged that it is primarily influenced by the interplay between endogenous and exogenous factors (Figure 1). Endogenous mechanisms encompass chronic low-grade inflammation, oxidative stress, mitochondrial dysfunction, hormonal alterations (such as reductions in testosterone, estrogen, vitamin D, DHEA, and IGF-1), apoptosis, and the decline in satellite cell function. Exogenous factors include a sedentary lifestyle, inadequate nutrition, obesity, and chronic diseases (Vitale et al., 2016; Dodds et al., 2017). These factors interact to disrupt the homeostatic regulation of skeletal muscle, ultimately leading to a decline in muscle mass and function.

Among the implicated mechanisms, mitochondrial dysfunction is considered one of the central factors driving muscle aging and the progression of sarcopenia (Ferri et al., 2020; Rygiel et al., 2016). Dysfunctional mitochondria continuously produce elevated levels of reactive oxygen species (ROS), which disrupt cellular redox balance and cause oxidative damage to proteins, lipids, and nucleic acids. This, in turn, affects the energy metabolism, functional maintenance, and survival capacity of skeletal muscle cells (Nemes et al., 2018; Speer and Mckune, 2021). Concurrently, the dysregulation of mitophagy exacerbates oxidative damage and metabolic disturbances, thereby establishing a self-perpetuating cycle. Additionally, “inflammaging” is regarded as a key contributing factor in sarcopenia (Antuña et al., 2022; Livshits and Kalinkovich, 2019). In elderly populations, pro-inflammatory cytokines (such as TNF-α, IL-1β, and IL-6) are persistently elevated, activating the NF-κB pathway, which promotes muscle protein degradation and inhibits protein synthesis, thereby accelerating muscle atrophy. Alterations in hormone levels also play a crucial role, as reductions in anabolic hormones like testosterone and IGF-1, coupled with the onset of insulin resistance, further impair muscle metabolism and synthesis (Meng and Yu, 2010; Chung et al., 2009). As individuals age, the number and differentiation potential of satellite cells gradually decrease, significantly impairing the regenerative and repair capacity of skeletal muscle (Sousa-Victor et al., 2022; Dumont et al., 2015). Furthermore, protein degradation pathways, including the ubiquitin-proteasome system (UPS) and autophagy, remain persistently activated during aging, leading to excessive degradation of structural proteins and aggravating muscle mass loss (Kelu and Hughes, 2025; Hughes et al., 2022).

In conclusion, the onset and progression of sarcopenia result from the combined effects of multiple mechanisms, including inflammatory responses, oxidative stress, metabolic imbalances, hormonal dysregulation, and stem cell depletion. A comprehensive understanding of these mechanisms not only elucidates the pathological basis of sarcopenia but also provides theoretical support for the development of potential therapeutic targets and the formulation of individualized intervention strategies.

### 2.3 Related signaling pathways

The onset and progression of sarcopenia are driven by the dysregulation of multiple key cellular signaling pathways, which are integral to the regulation of muscle synthesis, degradation, regeneration, and metabolism. These pathways form the foundation for contemporary molecular research in this domain.

The PI3K/Akt/mTOR pathway is a primary signaling cascade involved in the regulation of muscle protein synthesis. Its activation promotes muscle growth and regeneration. However, under sarcopenic conditions, this pathway is typically suppressed, leading to a reduction in protein synthesis and exacerbating muscle atrophy (Yoon, 2017; Bodine et al., 2001a). The AMPK pathway, which interacts with the PI3K/Akt/mTOR axis, functions as a cellular energy sensor. When energy levels are insufficient, AMPK is activated, promoting fatty acid oxidation, inhibiting protein synthesis, and maintaining metabolic balance. The dynamic equilibrium between AMPK and mTOR is crucial for sustaining skeletal muscle metabolic homeostasis (Egerman and Glass, 2014; Sandri, 2008). The NF-κB pathway, a key inflammation-associated transcription factor, is persistently activated in the state of “inflammaging,” resulting in the upregulation of pro-inflammatory cytokines such as TNF-α and IL-6. This, in turn, activates protein degradation pathways and inhibits protein synthesis, thereby contributing to the decline in muscle mass and function (Sriram et al., 2011; Fang et al., 2021). Similarly, the Myostatin/Smad pathway, a negative regulator of muscle growth, exhibits significantly elevated expression levels in elderly individuals, thereby inhibiting myofiber formation and satellite cell activity, positioning it as a potential target for therapeutic intervention (Mckay et al., 2012; Mccroskery et al., 2003).

Furthermore, the ubiquitin-proteasome system (UPS) and autophagy/mitophagy pathways are also critical in muscle protein degradation (Bodine et al., 2001b). The UPS, via E3 ubiquitin ligases such as MuRF1 and Atrogin-1, targets and degrades muscle structural proteins and is often found in a state of overactivation in the elderly. Dysregulation of the autophagy system, particularly impairments in mitochondrial clearance, leads to the accumulation of reactive oxygen species (ROS) and mitochondrial dysfunction, establishing a vicious cycle of oxidative stress and metabolic disturbances (Chen et al., 2023; Bodine and Baehr, 2014). Lastly, the Sirtuins/NAD^+^ pathway plays a pivotal role in aging and metabolic regulation, involving processes such as antioxidant defense, mitochondrial function maintenance, and DNA repair (Zhang et al., 2023). SIRT1 and SIRT3 are essential in regulating muscle metabolic homeostasis; however, their activity declines significantly with aging. NAD^+^, the required substrate for this pathway, is also considered a potential therapeutic target for delaying muscle aging (Gibril et al., 2024).

In conclusion, these signaling pathways collectively regulate various aspects of skeletal muscle homeostasis. Their dysregulation underpins the molecular basis of sarcopenia and provides multiple potential targets for the development of novel therapeutic interventions.

## 3 Pharmacological basis and pleiotropic effects of statins

### 3.1 Pharmacological actions and molecular mechanisms

Statins are a class of 3-hydroxy-3-methylglutaryl-CoA reductase (HMG-CoA reductase) inhibitors. They exert their primary effects by competitively inhibiting the activity of this enzyme, thereby blocking the rate-limiting step in the cholesterol biosynthesis pathway and effectively reducing plasma low-density lipoprotein cholesterol (LDL-C) levels. This mechanism forms the foundation for their therapeutic role in the primary and secondary prevention of cardiovascular diseases.

**FIGURE 2:**
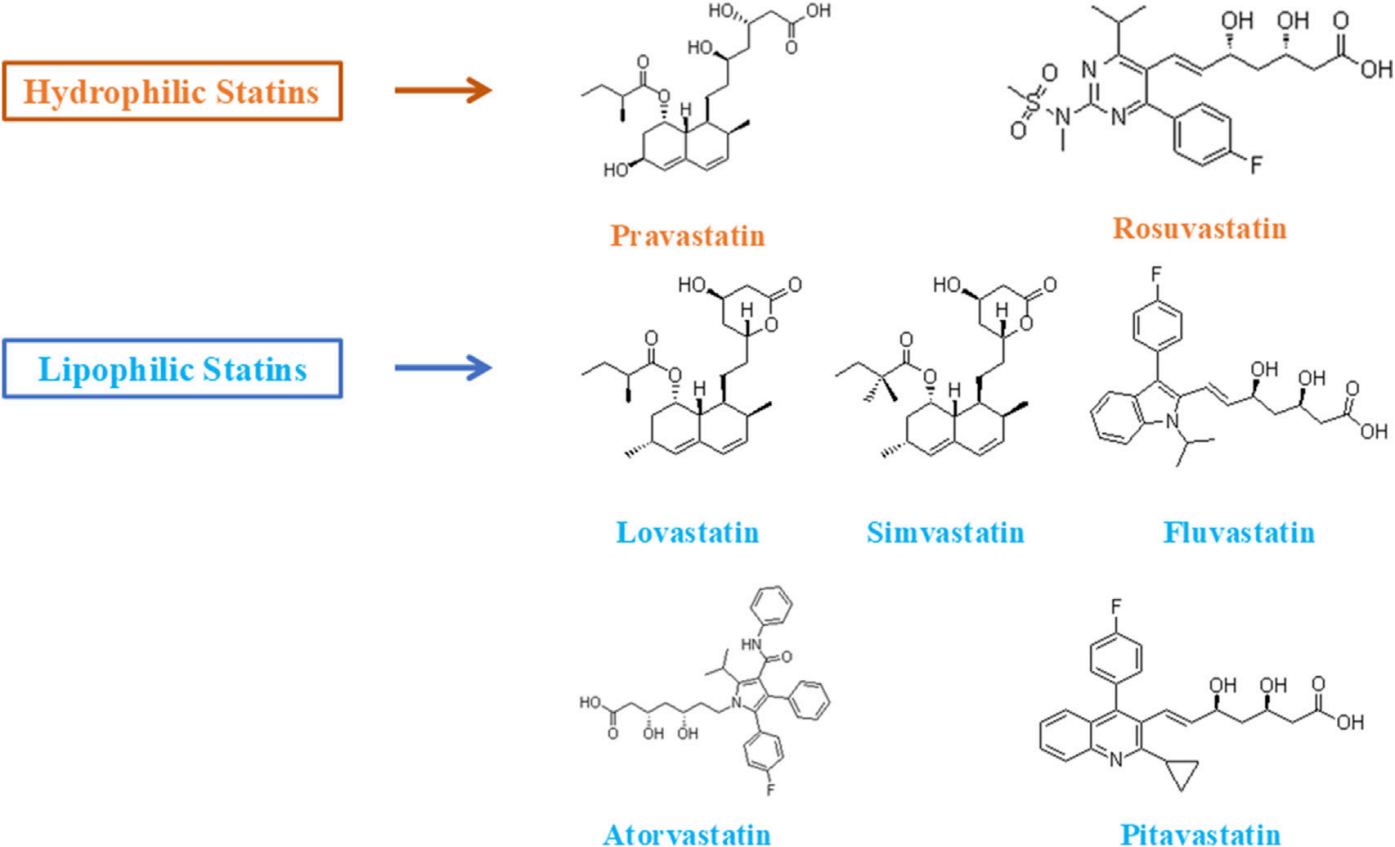
Lipophilic and hydrophilic statins.

From a molecular perspective, statins can be categorized into hydrophilic (e.g., pravastatin and rosuvastatin) and lipophilic (e.g., lovastatin, simvastatin, atorvastatin, and pitavastatin) classes (Figure 2). Hydrophilic statins enter hepatocytes through organic anion transporting polypeptides (OATPs) and primarily exert their effects in the liver. In contrast, lipophilic statins can cross cell membranes via passive diffusion and are more likely to accumulate in non-hepatic tissues, including skeletal muscle and bone tissue (Da Silva Pereira et al., 2025; Hsiang et al., 1999; Turner and Pirmohamed, 2019). Additionally, statins inhibit the mevalonate pathway, reducing the generation of several downstream metabolic intermediates such as isoprenoid compounds, coenzyme Q10, and Rho kinase activators (Di Stasi et al., 2010; Patel et al., 2022). This “cascade” metabolic intervention not only suppresses cholesterol synthesis but may also influence various physiological processes, including protein isoprenylation, mitochondrial function maintenance, cell signaling, and the regulation of skeletal muscle metabolism. Some studies suggest that statins may further activate the PI3K/Akt/mTOR signaling pathway by inhibiting the RhoA/Rho kinase (ROCK) pathway, thereby promoting protein synthesis, improving endothelial function, and exhibiting anti-inflammatory effects at low doses (Ahmadi et al., 2023). However, at high doses or in genetically predisposed individuals, the inhibition of coenzyme Q10 (CoQ10) synthesis and decreased mitochondrial membrane stability may induce energy metabolism disturbances and skeletal muscle cell apoptosis (Murtola et al., 2011). Evidence regarding this “dose-response relationship” primarily stems from *in vitro* experiments or animal models, and clinical studies have not reached consistent conclusions. One plausible hypothesis suggests that tissue distribution differences related to dose or the risk-benefit balance may form the physiological basis for this phenomenon, although the underlying mechanisms still require further investigation.

In conclusion, the effects of statins extend beyond their traditional lipid-lowering properties. By targeting multiple downstream molecular processes in the mevalonate pathway, statins influence key biological functions, including inflammation, oxidative stress, immune regulation, and skeletal muscle metabolism. These effects provide theoretical support for their potential applications in non-cardiovascular diseases, such as sarcopenia.

### 3.2 Anti-inflammatory and immune-modulatory effects

In addition to their lipid-lowering effects, statins have demonstrated substantial anti-inflammatory and immune-modulatory potential in various inflammation-related diseases. Importantly, these pleiotropic effects have been shown to be independent of their cholesterol-lowering actions (Satny et al., 2021). Chronic low-grade inflammation, often referred to as “inflammaging,” is considered a common pathological basis for a range of age-related diseases, including sarcopenia. The anti-inflammatory properties of statins provide novel intervention targets for potential therapeutic applications in these conditions (Jang et al., 2024).

At the cellular level, statins exert their anti-inflammatory effects by downregulating the NF-κB signaling pathway, thus inhibiting the inflammatory cascade. NF-κB, a key transcription factor, plays a central role in regulating the expression of pro-inflammatory cytokines, such as TNF-α, IL-1β, and IL-6. Numerous studies have demonstrated that statins can suppress the production of these cytokines and significantly reduce plasma C-reactive protein (CRP) levels, thus alleviating systemic low-grade inflammation (Razi et al., 2025; Hölschermann et al., 2006). The CARE and PRINCE studies have both confirmed that pravastatin reduces CRP levels without significantly lowering LDL-C, suggesting that its anti-inflammatory activity is independent of its lipid-lowering effects (Ridker et al., 1999; Albert et al., 2001).

Regarding immune modulation, statins can intervene in T cell activation and antigen presentation by inhibiting dendritic cell co-stimulatory molecules (such as CD80/86) and MHC-II-mediated presentation, thereby attenuating excessive immune responses (Ghittoni et al., 2006). Furthermore, certain studies suggest that statins regulate the Th17/Treg ratio, maintaining a dynamic balance between inflammatory and tolerogenic responses. In skeletal muscle and bone tissue, such immune modulation may help mitigate protein degradation, suppressed osteogenesis, and muscle atrophy induced by the inflammatory microenvironment (Chamani et al., 2023; Ma et al., 2020). Additionally, statins may indirectly inhibit the propagation of inflammation signals by improving endothelial function, enhancing the bioavailability of nitric oxide (NO), and downregulating the expression of adhesion molecules, thus stabilizing the local immune homeostasis of skeletal muscle and bone tissue (Greenwood and Mason, 2007; Woźniak et al., 2023).

In conclusion, statins modulate inflammation and immune processes through multiple mechanisms, including the inhibition of NF-κB signaling, downregulation of CRP and pro-inflammatory cytokine expression, and interference with T cell activation. These effects not only contribute to secondary prevention in cardiovascular diseases but also provide a robust molecular foundation for their translational application in inflammation-related degenerative diseases, such as sarcopenia.

### 3.3 Antioxidant and mitochondrial protective effects

Oxidative stress is recognized as one of the central pathological mechanisms underlying age-related degenerative diseases, including sarcopenia. As individuals age, mitochondrial function progressively deteriorates, leading to the excessive production and accumulation of reactive oxygen species (ROS), which disrupts cellular redox homeostasis. This, in turn, causes oxidative damage to proteins, lipids, and DNA, thereby accelerating muscle atrophy and cellular apoptosis (Hiona and Leeuwenburgh, 2008; Calvani et al., 2013). A substantial body of research indicates that the antioxidant defense effects of statins primarily involve the regulation of ROS generation and clearance, as well as the maintenance of mitochondrial function stability. Statins reduce ROS production by downregulating NADPH oxidase activity, and they also upregulate the expression of key antioxidant enzymes, such as superoxide dismutase (SOD) and catalase (CAT), thereby enhancing the cell’s ability to eliminate ROS (Margaritis et al., 2014; Adam and Laufs, 2008). Numerous *in vitro* and *in vivo* studies have demonstrated that atorvastatin and rosuvastatin can activate the Nrf2–ARE signaling pathway, inducing the expression of antioxidant-related genes, such as HO-1, GCLC, and NQO1, which further augment cellular antioxidant defense capacity (Wang et al., 2018; Li et al., 2020).

Furthermore, the mitochondrial protective effects of statins have attracted increasing attention. Under appropriate dosing conditions, statins stabilize mitochondrial membrane potential, maintain electron transport chain function, and reduce ROS leakage. Simultaneously, statins activate the AMPK–SIRT1–PGC-1α pathway, promoting mitochondrial biogenesis and repair, thereby delaying the functional decline of muscle cells (Khayatan et al., 2022; Singh et al., 2018). A large epidemiological study conducted in Denmark demonstrated that oxidative DNA damage markers, such as 8-oxodG, were significantly lower in statin users compared to non-users. This association was particularly pronounced in older individuals, as well as in those with hypertension and chronic kidney disease (Sørensen et al., 2019). However, it is important to emphasize that the antioxidant effects of statins are dose-dependent. High-dose exposure, particularly with lipophilic statins, may inhibit coenzyme Q10 (CoQ10) synthesis, thereby affecting the function of mitochondrial respiratory chain complexes I and III, ultimately inducing mitochondrial damage and muscle toxicity (Ryan et al., 2024; Rundek et al., 2004). Therefore, optimizing the dose and identifying individuals with vulnerable mitochondrial function are crucial prerequisites for ensuring the clinical safety of statins.

### 3.4 Molecular mechanisms of statins in sarcopenia

**FIGURE 3:**
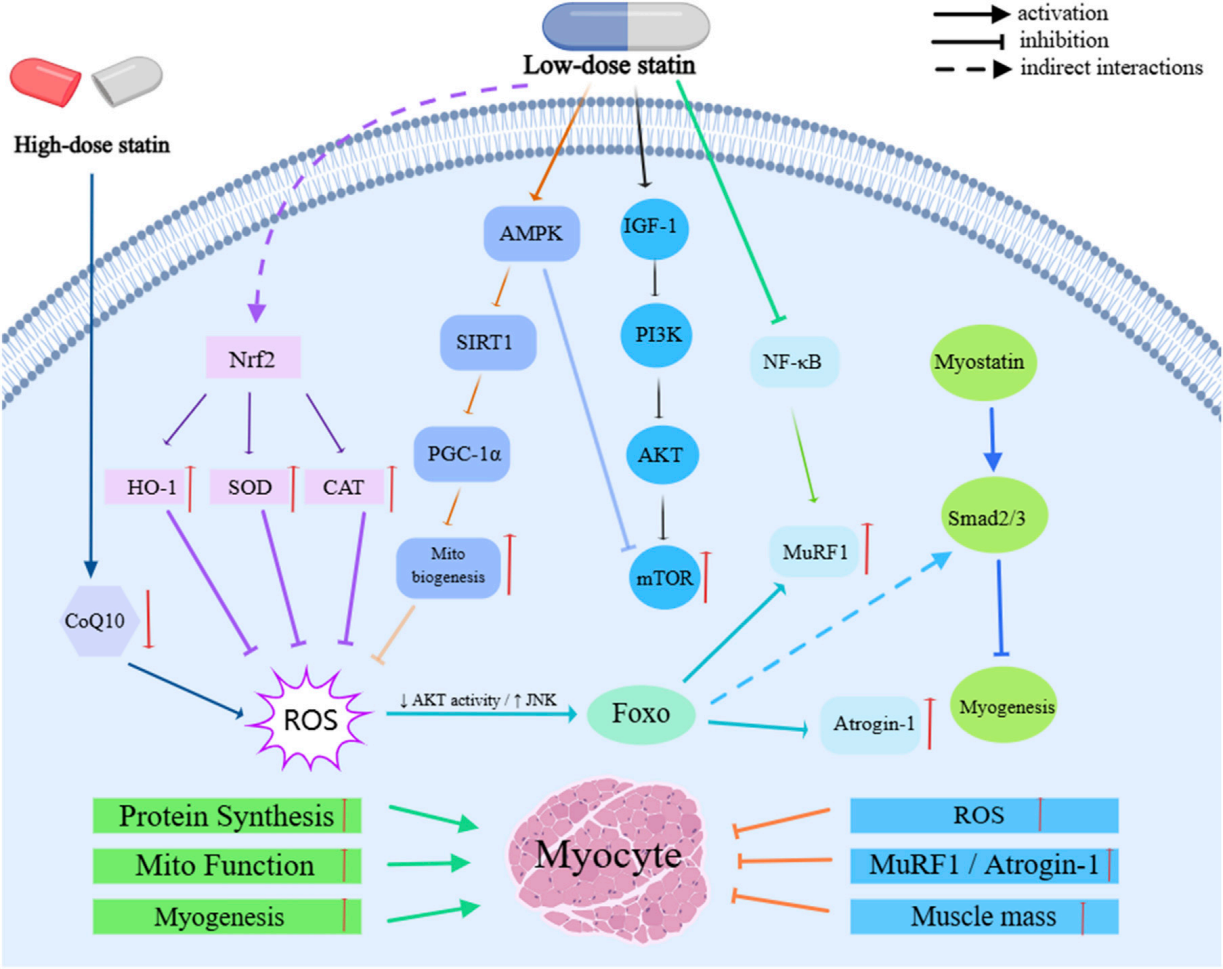
Molecular Mechanisms of Statins in sarcopenia.

The mechanisms by which statins exert their effects in sarcopenia involve several key molecular pathways, primarily focusing on four core aspects: “inflammation suppression—antioxidant effects—mitochondrial homeostasis—protein metabolism” (Figure 3).

By inhibiting the RhoA/ROCK axis and NF-κB signaling, statins downregulate the levels of pro-inflammatory cytokines, such as TNF-α, IL-1β, and IL-6, thereby alleviating chronic low-grade inflammation. This, in turn, inhibits protein degradation primarily via the ubiquitin-proteasome system (UPS), reducing myofiber atrophy and improving muscle function (Sriram et al., 2011; Fang et al., 2021; Razi et al., 2025; Hölschermann et al., 2006; Ridker et al., 1999; Albert et al., 2001). Additionally, statins activate the Nrf2–ARE pathway, which induces the expression of antioxidant genes, such as HO-1, NQO1, and GCLC, while simultaneously inhibiting NADPH oxidase activity. Statins also upregulate the expression of antioxidant enzymes like superoxide dismutase (SOD) and catalase (CAT), thereby reducing ROS accumulation and improving redox homeostasis, which mitigates the detrimental effects of oxidative stress on skeletal muscle (Hiona and Leeuwenburgh, 2008; Calvani et al., 2013; Margaritis et al., 2014; Adam and Laufs, 2008; Wang et al., 2018; Li et al., 2020). At appropriate doses, statins further activate the AMPK–SIRT1–PGC-1α pathway, promoting mitochondrial biogenesis and repair, stabilizing mitochondrial membrane potential, and maintaining electron transport chain function. This reduces ROS leakage and inhibits mitochondrial-dependent apoptosis. However, high-dose exposure or use in susceptible individuals may lead to mitochondrial dysfunction due to the inhibition of coenzyme Q10 (CoQ10) synthesis, thereby increasing the risk of muscle toxicity (Khayatan et al., 2022; Singh et al., 2018; Sørensen et al., 2019; Ryan et al., 2024; Rundek et al., 2004).

Moreover, statins play a crucial role in regulating protein metabolism. They enhance the PI3K/Akt/mTOR signaling pathway to promote protein synthesis and downregulate key UPS E3 ligases, such as MuRF1 and Atrogin-1. Statins also moderately activate autophagy and mitophagy, facilitating the clearance of damaged proteins and mitochondria, thereby maintaining skeletal muscle homeostasis and function (Bodine et al., 2001b; Chen et al., 2023; Bodine and Baehr, 2014). At the microcirculatory level, statins improve muscle perfusion and oxygen supply by inhibiting the RhoA/ROCK pathway and upregulating endothelial nitric oxide synthase (eNOS), thereby supporting tissue repair. Additionally, their interaction with the Myostatin/Smad pathway further regulates satellite cell activity (Mckay et al., 2012; Mccroskery et al., 2003; Greenwood and Mason, 2007; Woźniak et al., 2023). These effects are modulated by multiple factors, including dose, drug physicochemical properties, and individual background. Lower doses tend to provide protective effects, while higher doses may shift the drug’s action into a detrimental window. Lipophilic formulations, which are more likely to accumulate in muscle tissue, exhibit stronger effects and higher risks (Da Silva Pereira et al., 2025; Hsiang et al., 1999; Turner and Pirmohamed, 2019; Ahmadi et al., 2023; Murtola et al., 2011). Furthermore, individual genetic factors—particularly SLCO1B1 polymorphisms—can influence drug exposure and muscle toxicity risk, ultimately determining both efficacy and safety (Carr et al., 2013; Xiang et al., 2018).

In conclusion, statins regulate redox homeostasis and maintain the balance of mitochondrial and protein metabolism through multiple molecular mechanisms. These effects offer potential benefits in delaying age-related muscle degeneration, providing a robust biological and pharmacological foundation for their translational application in sarcopenia.

## 4 Research progress on the role of statins in the prevention and treatment of sarcopenia

**FIGURE 4:**
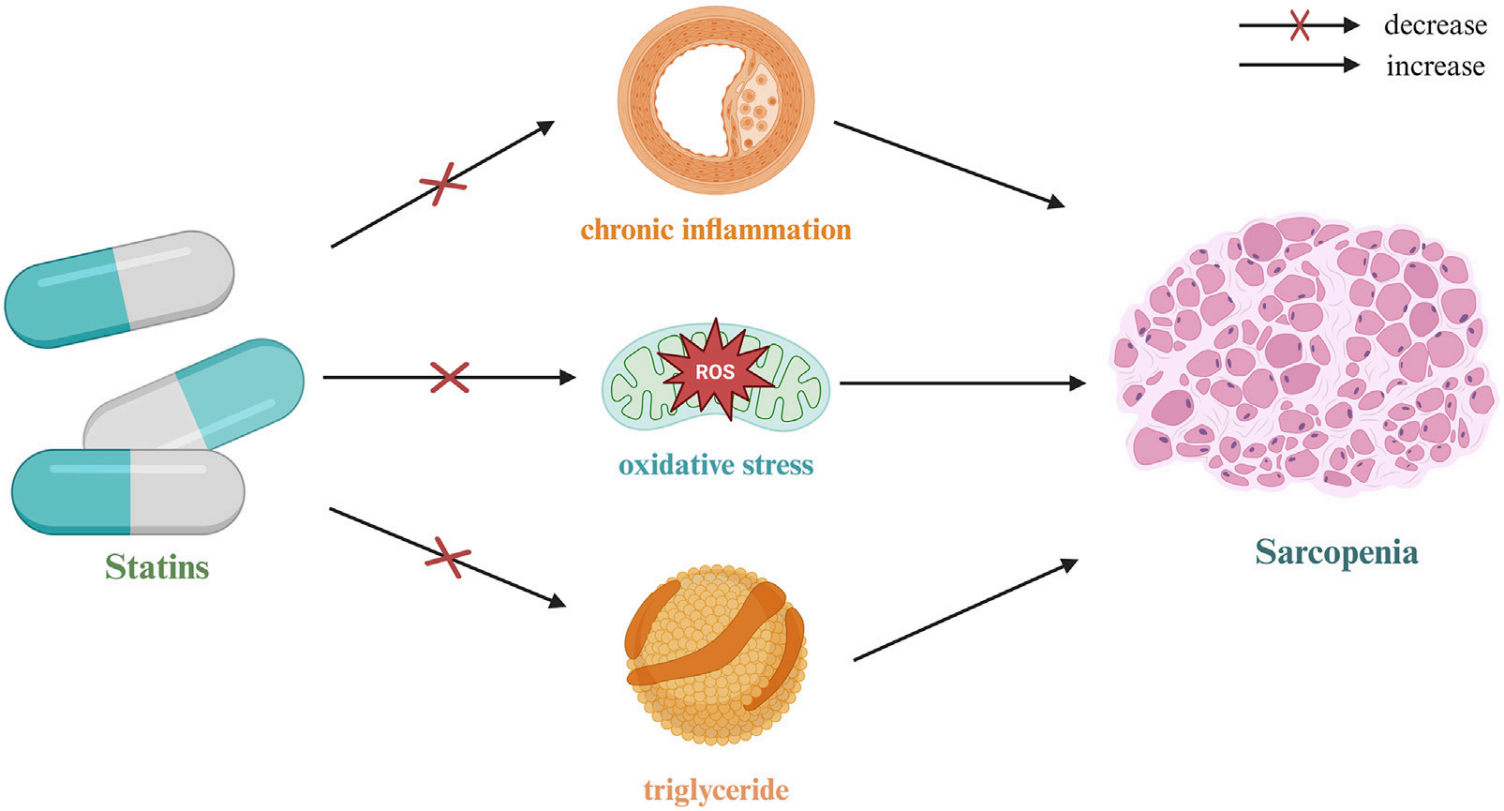
Mechanism of action of statins in sarcopenia.

Over the past decade, the growing body of research on the pleiotropic effects of statins has prompted scholars to shift their focus from the traditional lipid-lowering function to the broader potential of statins in various biological processes, particularly in inflammation suppression, antioxidation, immune modulation, and microcirculation improvement. Sarcopenia, as a core syndrome associated with aging, has garnered increasing attention in recent years. As research progresses, scholars have begun investigating whether statins can delay the decline in skeletal muscle mass and function through their multifaceted mechanisms (Figure 4). Preliminary results indicate that, within a specific dose range, statins may serve as a potential intervention for sarcopenia by improving muscle metabolic homeostasis, inhibiting the degradation of inflammation-related proteins, and enhancing mitochondrial function.

### 4.1 Progress in research on anti-inflammatory mechanisms in sarcopenia models

Chronic low-grade inflammation is considered a central driving factor in the onset and progression of sarcopenia, particularly among the elderly, in whom the inflammatory state is often continuously activated. Statins alleviate the inflammatory stimuli in the skeletal muscle microenvironment by downregulating the expression of pro-inflammatory cytokines, such as TNF-α, IL-6, and IL-1β, through inhibition of the NF-κB signaling pathway (Ortego et al., 1999). Studies have demonstrated that NF-κB activation not only enhances the activity of protein degradation pathways (such as the ubiquitin-proteasome system, UPS) but also inhibits protein synthesis, thereby further accelerating muscle loss (Jackman and Kandarian, 2004). A large-scale prospective clinical study revealed that pravastatin significantly reduced CRP levels without significantly lowering LDL-C, indirectly confirming the independence of its anti-inflammatory effects (Ridker et al., 1999). Animal studies also demonstrated that low-dose statin intervention effectively reduced myofiber atrophy and mitigated the infiltration of inflammatory cells, suggesting its potential role in maintaining immune homeostasis (Zheng et al., 2023).

### 4.2 Evidence for antioxidant mechanisms in the intervention of age-related muscle dysfunction

As individuals age, skeletal muscle tissue is continuously subjected to oxidative stress. The accumulation of reactive oxygen species (ROS) not only damages mitochondrial DNA and muscle structural proteins but also activates FoxO transcription factors, thereby enhancing the activity of protein degradation pathways (Pomiès et al., 2016; Dodd et al., 2010). Numerous foundational studies have demonstrated that statins, by activating the Nrf2/ARE pathway, induce the expression of antioxidant enzymes (such as HO-1 and GCLC), thereby enhancing the cell’s capacity to eliminate ROS and mitigating the cellular damage caused by oxidative stress (Kwok et al., 2012; Mansouri et al., 2022). An epidemiological study involving 19,795 participants revealed a significant association between statin use and a reduction in oxidative DNA damage markers, such as 8-oxodG, with particularly pronounced effects in the elderly population (P_interaction <0.001). This provides indirect, population-based evidence for the muscle-protective effects of statins (Sørensen et al., 2019). Additionally, animal experimental evidence shows that statins can increase the expression of superoxide dismutase (SOD) and catalase (CAT) in skeletal muscle, inhibit lipid peroxidation, and thereby improve muscle functional status (Wu et al., 2016).

### 4.3 Modulation of skeletal muscle blood flow and mitochondrial homeostasis

In addition to inflammation and oxidative stress, the development of sarcopenia also involves pathological processes such as inadequate skeletal muscle blood flow and mitochondrial dysfunction. Research has shown that statins improve microcirculatory perfusion in skeletal muscle by enhancing endothelial function, upregulating eNOS expression, and increasing the bioavailability of nitric oxide (NO), thereby enhancing oxygen and nutrient supply and improving metabolic status (Mason et al., 2015). Studies have indicated that, at moderate doses, statins can regulate mitochondrial membrane potential and oxidative phosphorylation, while also promoting the expression of PGC-1α, thus maintaining mitochondrial function stability and reducing ROS generation. Animal experiments further demonstrate that moderate doses of simvastatin or rosuvastatin can enhance ATP synthesis efficiency, inhibit the activation of apoptosis-related signals (such as cytochrome c release and caspase-9/3 cascade activation), thereby exerting mitochondrial protective effects (Ramachandran and Wierzbicki, 2017).

### 4.4 Exploration of synergistic or antagonistic effects between statins and exercise/nutritional interventions

In clinical practice, the standard interventions for sarcopenia primarily consist of exercise training and nutritional support (e.g., supplementation with protein, vitamin D, β-hydroxy-β-methylbutyrate [HMB], etc.). The potential for statins to synergize with these interventions or, conversely, interfere with their effects remains an unresolved issue in current research. Some studies suggest that statins may synergistically enhance the effects of aerobic exercise by activating the PI3K/Akt/mTOR pathway, thereby increasing muscle protein synthesis rates. Furthermore, combining statins with vitamin D may improve muscle strength and reduce inflammation (Fadah et al., 2025). However, other studies indicate that high-dose statins could interfere with muscle adaptation to exercise, leading to adverse outcomes such as muscle pain and delayed recovery. Notably, these side effects may be exacerbated in the context of exercise-induced ROS elevation (Ryan et al., 2024). Therefore, when implementing combined intervention strategies, it is essential to consider individual factors such as age, exercise capacity, and baseline inflammatory status, as well as to perform dynamic risk assessments to optimize personalized intervention plans.

In conclusion, statins may exert a beneficial role in the prevention and treatment of sarcopenia through multiple mechanisms, including the regulation of inflammation, alleviation of oxidative stress, improvement of blood flow and mitochondrial function, and potential synergy with traditional interventions such as exercise and nutritional supplementation. While existing basic and clinical studies provide preliminary evidence supporting their potential value, the therapeutic efficacy and safety of statins require further validation through large-scale, standardized dose studies and long-term prospective clinical trials.

## 5 Summary and evaluation of statins and their role in sarcopenia

In recent years, the potential impact of statins on the risk of sarcopenia has attracted considerable attention. On one hand, some studies suggest that statins may exert protective effects on skeletal muscle health through pleiotropic mechanisms, including anti-inflammatory, antioxidant, and microcirculatory improvements. On the other hand, there is also evidence indicating that, in certain populations, these drugs may exacerbate muscle toxicity. Given the significant heterogeneity in the research findings, these differences may be closely related to factors such as drug type, dosage, subject characteristics, and study design. Therefore, a systematic review of the current evidence is needed. This article will comprehensively analyze the existing research from three perspectives: types of study results, mechanisms of individual differences, and research limitations (Table 1).

**TABLE 1:** Summary of clinical studies evaluating statin use and its impact on sarcopenia risk and outcomes.

Study Type	Statin Type	Number of Cases	Subject and Population	Outcome Measures	Refined Conclusion	Association with Sarcopenia	References
RS	Not reported	136 (25 Sarcopenia)	Heart failure (HF) patients (HF outpatients)	Multivariable OR for sarcopenia	Statins inversely associated with sarcopenia	Potentially Protective	Valdiviesso et al. (2022)
RS	Not reported	136 (25 Sarcopenia)	Heart failure (HF) patients (HF outpatients)	Multivariable OR for sarcopenia	Statins inversely associated with sarcopenia	Potentially Protective	Valdiviesso et al. (2023)
RS	Atorvastatin, pitavastatin, fluvastatin and simvastatin	322 (90 Sarcopenia)	Patients with cardiovascular diseases (CVD inpatients)	Muscle mass comparison	Statin use linked to better muscle mass	Potentially Protective	Harada et al. (2017)
RS	Not reported	3,422 (690 on statin)	Dundee for inpatient rehabilitation (Rehabilitation inpatients)	Barthel score improvement	Greater functional improvement with statins	Potentially Protective	Lynch et al. (2012)
RCT	Pitavastatin	510 (254 on statin)	People with HIV (HIV+ individuals)	Muscle area, muscle density (via MRI), clinical outcomes	Pitavastatin had no impact on muscle mass	No Significant Association	Erlandson et al. (2025)
RS	Not reported	756 (582 on statin)	Patients with abdominal aortic aneurysm (AAA)	Skeletal muscle index (SMI), muscle density (SMD)	Statins in AAA patients did not impact muscle mass	No Significant Association	Bradley et al. (2025)
RS	Pravastatin, fluvastatin, simvastatin, atorvastatin, rosuvastatin, pitavastatin and cerivastatin	1,636 (342 on statin)	Community-dwelling adults (Japan) (Middle-aged & older adults)	No sig. association	Statins not linked to sarcopenia	No Significant Association	Huang et al. (2025)
CCS	pravastatin, fluvastatin, rosuvastatin, simvastatin, lovastatin and atorvastatin	67,001 (17,634 on statin)	Patients with chronic kidney disease (CKD patients)	Prevention of sarcopenia	Statins may reduce sarcopenia risk	No Significant Association	Lin et al. (2020)
RS	Atorvastatin, rosuvastatin, simvastatin, and fluvastatin	216 (119 on statin)	AAA patients (EVAR) (Post-EVAR)	No increase in sarcopenia	No sarcopenia increase	No Significant Association	Lindström et al. (2021)
CS	Atorvastatin, simvastatin, rosuvastatin and pravastatin	366 (53 on statin)	People with HIV (HIV+ individuals)	No worsening of muscle metrics	No exacerbation of muscle wasting	No Significant Association	Cárdenas et al. (2023)
CCS	Not reported	93 (14 Sarcopenia)	Renal transplant recipients (Kidney transplant recipients)	Statin = risk factor	Statins as a risk factor	Potentially Detrimental	Ozcan et al. (2024)
RW and GWAS	Atorvastatin, simvastatin and rosuvastatin	4,107 (33 Sarcopenia)	FAERS dataset (Mixed (pharmacovigilance))	PRR = 4.68, χ² = 903	Atorvastatin among top 5 sarcopenia drugs	Potentially Detrimental	Zhang and Yao (2024)
RS	Not reported	586 (241 Sarcopenia)	Stroke patients (Post-stroke patients)	Reduced strength recovery	Negative impact on strength recovery	Potentially Detrimental	Matsumoto et al. (2023)
CCS	Not reported	172 (50 on statin)	CHF patients (Chronic HF)	NMJ & gut barrier damage	Systemic inflammation and disability	Potentially Detrimental	Ahmad et al. (2023)
CS-CC	Not reported	152 (75 on statin)	Older adult men (Older adult males)	Reduced SarQoL & NMJ degeneration	NMJ degradation and QoL decline	Potentially Detrimental	Qaisar et al. (2024)

RCT, randomized controlled trial; RS, retrospective study; CCS, case-control study; CS, cross-sectional study; RW and GWAS, real-world and GWAS study; CS-CC, cross-sectional case-control study.

### 5.1 Potential protective effects of statins

Several observational and retrospective studies suggest that statins may offer a protective effect on muscle mass and function to some extent. Specifically, a cross-sectional study found a negative correlation between statin use and sarcopenia (OR = 0.03; 95% CI: 0.01–0.30), hypothesizing that statins may help maintain neuromuscular homeostasis by reducing the levels of pro-inflammatory cytokines, increasing the bioavailability of nitric oxide (NO), and improving endothelial function (Valdiviesso et al., 2022). Similar findings were observed in heart failure patients, where statin use was significantly associated with a lower risk of sarcopenia (Valdiviesso et al., 2023). Another retrospective analysis revealed that patients receiving statin treatment had significantly higher skeletal muscle mass and skeletal muscle index (SMI) than those who did not (Harada et al., 2017). Moreover, a 10-year cohort follow-up study demonstrated that elderly patients in the statin group showed greater improvements in the Barthel index compared to non-users (3.59 vs. 4.30, P < 0.001), suggesting that statins may have a positive impact on functional recovery in the elderly population (Lynch et al., 2012).

In summary, the available research preliminarily supports the potential muscle-protective effects of statins in certain elderly populations or patients with cardiovascular disease. However, due to the observational nature of many of these studies, along with limitations such as population heterogeneity and inconsistent dosages, the generalizability and causal inference of the current evidence must be interpreted with caution.

### 5.2 Studies showing no significant association or neutral effects

Certain research findings suggest that statin use does not exhibit a significant association with sarcopenia, indicating a potential “neutral effect” in specific populations. A recent study, the REPRIEVE trial (n = 510), demonstrated that after 24 months of pitavastatin use in individuals with HIV (PWH), no significant changes in muscle mass were observed, suggesting that pitavastatin did not have an adverse impact on PWH (Erlandson et al., 2025). Additionally, a study involving patients with abdominal aortic aneurysms (n = 150) revealed that long-term statin use did not significantly affect muscle mass or density, with no notable effects on muscle observed (Bradley et al., 2025). Another prospective cohort study from Japan (n = 1636) found no statistical association between statin use and declines in muscle mass, strength, or function. The results remained robust after multivariate adjustment and sensitivity analysis (Huang et al., 2025). In a long-term follow-up study of patients with chronic kidney disease, no significant association was found between statin use and the risk of sarcopenia, with some analyses even suggesting a mild protective trend (Lin et al., 2020). Furthermore, in patients who underwent endovascular aneurysm repair (EVAR) and individuals with HIV, no significant adverse effects on skeletal muscle were observed, with some subgroups even showing trends of functional improvement (Lindström et al., 2021; Cárdenas et al., 2023).

In conclusion, these findings suggest that, in certain underlying disease contexts, statins may exert a neutral or mildly protective effect on skeletal muscle. However, such conclusions should be interpreted cautiously, considering factors such as population characteristics, underlying disease status, and dosage.

### 5.3 Potential increased risk in specific populations

Compared to the general population, certain high-risk individuals are at a significantly higher risk of experiencing adverse muscle reactions following statin use. A study involving kidney transplant recipients identified statin use as an independent risk factor for sarcopenia (OR = 1.34, P = 0.02) (Ozcan et al., 2024). Another real-world data-based signal detection study revealed a significant association between atorvastatin use and the incidence of sarcopenia (PRR = 4.68, χ^2^ = 903) (Zhang and Yao, 2024). In stroke rehabilitation patients, statin use was significantly associated with delayed muscle strength recovery (β = −0.41, P < 0.01) (Matsumoto et al., 2023). Additionally, in patients with chronic heart failure, research suggests that statins may induce muscle inflammation and functional decline by interfering with neuromuscular junction function and intestinal barrier stability (Bielecka-Dabrowa et al., 2018; Ahmad et al., 2023). A cross-sectional study further demonstrated that statin users exhibited poorer performance in muscle strength and quality of life scores compared to non-users (Qaisar et al., 2024).

In summary, the available evidence indicates that in specific clinical settings—such as kidney transplantation, post-stroke rehabilitation, or severe heart failure—especially when high doses or lipophilic statins are used, careful consideration of the potential risks and benefits is essential to prevent unnecessary muscle toxicity.

### 5.4 Clinical data summary and comprehensive evaluation

A comprehensive analysis of the existing clinical research data reveals significant heterogeneity in the role of statins in sarcopenia. Specifically, some studies suggest that statins may offer a degree of protection to skeletal muscle through pleiotropic mechanisms such as anti-inflammatory, antioxidant, and vascular health improvement (Valdiviesso et al., 2022; Valdiviesso et al., 2023; Harada et al., 2017; Lynch et al., 2012). Although these observational studies indicate potential benefits of statins in elderly populations or individuals with cardiovascular diseases, establishing causal relationships remains challenging due to the observational nature of the research design and the heterogeneity within the studied populations. On the other hand, some studies have found no significant association between statins and sarcopenia, and even suggest that statin use may have a mild protective effect on muscle in certain chronic disease patients. This observation suggests that the efficacy of statins may vary depending on individual differences across patient backgrounds (Erlandson et al., 2025; Bradley et al., 2025; Huang et al., 2025; Lin et al., 2020; Lindström et al., 2021; Cárdenas et al., 2023). It is important to note that most of these studies are long-term follow-up studies, and the results remain stable even after adjusting for multiple variables. However, some studies suggest that certain high-risk populations—such as kidney transplant recipients and stroke rehabilitation patients—may experience a significantly increased risk of adverse muscle reactions when using statins, particularly when high doses or lipophilic statins are used (Ozcan et al., 2024; Zhang and Yao, 2024; Matsumoto et al., 2023; Bielecka-Dabrowa et al., 2018; Ahmad et al., 2023; Qaisar et al., 2024). In kidney transplant patients, statins may increase the risk of sarcopenia by altering muscle metabolism and immune responses.

Thus, existing clinical trials and research indicate that when using statins for the treatment of sarcopenia, factors such as dosage, drug type, and the patient’s individual background are key determinants of efficacy and safety. Based on the available evidence, although some studies support the potential muscle-protective effects of statins in certain elderly populations or patients with cardiovascular diseases, caution is necessary in high-risk populations, particularly when high doses or lipophilic statins are used. Personalized treatment strategies should be implemented to optimize outcomes.

### 5.5 Individual susceptibility and pharmacogenetic explanations

In recent years, individual susceptibility and genetic variations related to drug metabolism have garnered considerable attention as explanations for the inconsistencies observed in the conclusions of existing studies. Specifically, the C521T polymorphism in the SLCO1B1 gene has been shown to significantly influence the uptake and metabolic efficiency of lipophilic statins in the liver, leading to increased drug concentrations and a corresponding elevation in the risk of muscle toxicity. Individuals carrying this genetic mutation are more predisposed to experiencing adverse effects such as muscle pain, muscle weakness, and even severe reactions like rhabdomyolysis (Carr et al., 2013; Xiang et al., 2018). Consequently, pharmacogenomics-guided therapy, which integrates pharmacogenomic data to tailor statin treatment to individual profiles, is considered a promising approach to optimizing therapeutic efficacy while minimizing adverse effects. In the future, the application of pharmacogenomics is anticipated to play a pivotal role in enhancing the effectiveness of drug interventions in sarcopenia.

### 5.6 Limitations and challenges in current research

While research on the relationship between statins and sarcopenia has increased in recent years, the existing preliminary evidence provides valuable theoretical insights into underlying mechanisms and clinical trends. However, from the standpoint of study design and evidence quality, several limitations remain. First, the majority of studies are retrospective observational or cross-sectional in nature, lacking high-quality randomized controlled trials (RCTs), which limits the ability to establish clear causal relationships. Moreover, many studies feature relatively short follow-up periods, and the outcome measures employed are often not standardized, which restricts comprehensive evaluations of long-term efficacy and safety. Second, significant heterogeneity exists in key variables across studies, including drug type, dosage, and duration of intervention. For instance, hydrophilic and lipophilic statins exhibit substantial differences in tissue distribution and metabolic pathways, yet most studies lack adequate subgroup comparisons. Furthermore, variations in treatment doses and durations across studies severely compromise the comparability and generalizability of the results. In some studies, dosage grading standards for statins are unclear, and the methods for calculating dose intensity are inconsistent, further hindering the ability to quantify dose-response relationships and integrate meta-analyses. Finally, most current studies have not sufficiently controlled for individual factors such as age, sex, baseline muscle status, nutritional levels, comorbidities (e.g., chronic kidney disease, diabetes), exercise capacity, and genetic backgrounds (e.g., SLCO1B1 polymorphisms). These factors may significantly influence drug metabolism and its effects on the muscle system, representing key contributors to the observed heterogeneity in study outcomes. Therefore, future research should consider integrating pharmacogenomic characteristics, physiological indicators, and inflammatory/metabolic biomarkers within a stratified population model, to develop a “dose-response-individual background” approach. Such an approach would facilitate the clinical translation and practical application of precision medicine strategies in the management of sarcopenia.

## 6 Summary and outlook

Sarcopenia is a degenerative disorder intrinsically linked to the aging process, particularly prevalent among the elderly population globally. It significantly impairs both quality of life and individual independence. Currently, the primary strategies for managing sarcopenia rely on exercise regimens and nutritional support. However, the effectiveness of these interventions is often limited, particularly for frail individuals or those suffering from multiple chronic conditions. Consequently, there is an urgent need for the development of novel pharmacological interventions.

In recent years, statins, renowned for their pleiotropic biological effects, have garnered attention for their potential benefits in promoting skeletal muscle health, due to their anti-inflammatory, antioxidant, and microcirculation-enhancing properties. Some studies suggest that within an appropriate dosage range, statins may help improve muscle metabolic status and delay functional decline. However, other studies indicate that at higher doses or in genetically predisposed individuals, statins may induce mitochondrial dysfunction, inhibit coenzyme Q10 (CoQ10) synthesis, and increase the risk of adverse muscular side effects. This phenomenon suggests that the relationship between statins’ dosage and their effects may be non-linear, necessitating careful consideration of individual variability when applying these treatments in clinical settings.

Based on the available evidence, this paper proposes a simplified “dose–mechanism–individual background” interaction model: low doses of statins may exert protective effects through mechanisms such as anti-inflammatory, antioxidative, and metabolic enhancement, while higher doses may increase the risk of muscle toxicity through mechanisms like mitochondrial damage. Furthermore, differences in tissue distribution and metabolic pathways between lipophilic and hydrophilic statins, along with individual genetic profiles (e.g., SLCO1B1 gene polymorphisms) and metabolic states, may collectively influence the efficacy and safety of statins.

Future research should focus on several critical areas: First, the establishment of a clear dose–effect–toxicity relationship model is essential to explore the mechanisms of action of different statin types and doses on skeletal muscle health. Second, large-scale, stratified prospective clinical studies are needed to systematically evaluate the efficacy and safety of statins across diverse populations. Third, the integration of pharmacogenomics and metabolic biomarkers should be prioritized to support the development of personalized, precision medicine strategies. Finally, further investigations are required to elucidate the synergistic mechanisms between statins and traditional interventions, such as exercise and nutritional supplementation, to optimize comprehensive treatment plans and enhance clinical outcomes.

In conclusion, although the potential for statins in the management of sarcopenia remains in its exploratory phase, it is crucial to combine mechanistic studies with clinical validation to foster their translational application in muscle health management, particularly in the context of aging. This will open up new pharmacological avenues for intervention in relevant populations.
